# Detection of *In Vivo* Inflammasome Activation for Predicting Sepsis Mortality

**DOI:** 10.3389/fimmu.2020.613745

**Published:** 2021-02-04

**Authors:** Jing Cui, Stephanie Oehrl, Fareed Ahmad, Thorsten Brenner, Florian Uhle, Christian Nusshag, Christoph Rupp, Felix Funck, Stefan Meisel, Markus A. Weigand, Christian Morath, Knut Schäkel

**Affiliations:** ^1^ Department of Dermatology, Heidelberg University Hospital, Heidelberg, Germany; ^2^ Department of Dermatology, Affiliated Hospital of Guizhou Medical University, Guiyang, China; ^3^ Department of Anesthesiology and Intensive Care, University Hospital Essen, Essen, Germany; ^4^ Department of Anesthesiology, Heidelberg University Hospital, Heidelberg, Germany; ^5^ Department of Nephrology, Heidelberg University Hospital, Heidelberg, Germany

**Keywords:** inflammasome, sepsis, monocytes, biomarker, ASC-speck

## Abstract

Sepsis is a severe life-threatening syndrome caused by dysregulated host responses to infection. Biomarkers that allow for monitoring the patient’s immune status are needed. Recently, a flow cytometry-based detection of *in vivo* inflammasome activation by formation of cytoplasmic aggregates of ASC (apoptosis-associated speck-like protein containing a caspase recruitment domain) has been proposed. Here we report on the frequency of ASC-speck^+^ leukocytes correlating with the survival of sepsis. 25 patients with sepsis were sampled consecutively for 7 days. Blood, serum samples and patient data were collected according to the guidelines of the PredARRT-Sep-Trial. Flow cytometric analysis was performed on fresh whole blood samples to investigate the formation of ASC-specks in leukocyte subsets. Serum samples were analyzed for production of IL-1ß, IL-18 and additional inflammatory markers. ASC-speck formation was found to be increased in leukocytes from sepsis patients compared to healthy donor controls. The absolute number of ASC-speck^+^ neutrophils peaked on day 1. For monocytes, the highest percentage and maximum absolute number of ASC-speck^+^ cells were detected on day 6 and day 7. Inflammatory cytokines were elevated on day 1 and declined thereafter, with exception of IL-18. Survival analysis showed that patients with lower absolute numbers of ASC-speck^+^ monocytes (<1,650 cells/ml) on day 6 had a lower probability to survive, with a hazard ratio (HR) of 10.178. Thus, the frequency of ASC-speck^+^ monocytes on day 6 after onset of sepsis may serve to identify patients at risk of death from sepsis.

## Introduction

Sepsis remains a major cause of mortality in intensive care units (ICU) worldwide (1–3). The dysregulated host responses in sepsis allows for excessive inflammation and immunosuppression (4). A hyper-inflammatory response predominates at the early stage, anti-inflammatory mechanisms manifest concomitantly, leading to long-term immune suppression. Owing to advances in care, most patients with sepsis survive the initial phase. However, if sepsis persists, patients enter a protracted hypo-inflammatory phase with significant immunosuppression (5–9) and the majority of non-surviving patients die in this phase (9–11). Failure in controlling the primary infection or the acquisition of secondary infections, caused by opportunistic bacteria and fungi, are the major cause of death during this period (10, 12). The risk of late-deaths from sepsis appears to be directly related to the immunosuppressive state of leukocytes (13, 14).

Monitoring the immune status of sepsis patients and the application of biomarker-guided interventions are needed for designing future therapeutic interventions allowing protection of these patients from late death (13). Biomarkers identifying patients at risk are largely missing (15). Inflammasome activation is of central importance for immune defense against infection and is a key component of sepsis pathophysiology (16–21). Inflammasomes are large protein complexes formed on encounter of microbial or damage associated stimuli (22). They regulate at least two host responses during sepsis: maturation and secretion of the pro-inflammatory cytokines interleukin-1β (IL-1β) as well as IL-18 and induction of pyroptosis, a rapid lytic form of programmed cell death (23–25). Central to the function of most inflammasome structures is the adapter molecule apoptosis-associated speck-like protein containing a caspase-recruitment domain (ASC). When inflammasomes are activated and assembled, ASC relocalizes from its diffuse cytoplasmatic distribution at steady state into a single speck, serving as a supramolecular signaling platform. This is followed by caspase-1 activation, thereby, cleaving the pro-peptide to generate mature and bioactive IL-1β and IL-18. The aberrant activation of inflammasomes has been reported in several autoimmune and chronic inflammatory diseases, such as cryopyrin-associated periodic syndrome (CAPS), familial mediterranean fever (FMF), gout and asthma. However, little work has been devoted to the role of inflammasomes in acute inflammatory diseases. Lee et al. reported that downregulation of nucleotide-binding and oligomerization domain-like receptor (NLR) family pyrin domain (PYD)containing 3 (NLRP3) inflammasome activation leads to increased survival in a polymicrobial sepsis mouse model (17). Another study found that NLRP3 inflammasome knockout mice were more susceptible to *S. schenckii* infection than wild-type mice, suggesting that inflammasomes contribute to host protection as well (26). Caspase-1 was shown to predict the outcome of sepsis (27–29), in that high caspase-1 activation during the first day of sepsis correlated with poor sepsis outcome (29). However, little work has been devoted to the role of ASC, the upstream process of caspase-1, in sepsis. The *in vivo* ASC-speck formation within blood leukocytes is a distinguishing feature of inflammasome formation and permits a direct and quantitative study of blood samples by flow cytometry. This assay was well validated (30–32) and has been implemented previously (33, 34).

Given the potential relevance of *in vivo* ASC-speck formation as a biomarker of systemic immune activation, we studied ASC-speck formation in leukocytes of sepsis patients during the first week after onset. Here, we report on a flow cytometry-based detection of ASC-specks in monocytes serving as a potential biomarker of patients at risk of death from sepsis.

## Methods

### Sample Collection

We report data of 141 samples from 25 patients with sepsis or septic shock, representing a subgroup of patients participating in the PredARRT-Sep-Trial (DRKS-ID: DRKS00012446) (35), hospitalized in the surgical ICU of Heidelberg University Hospital from September 2017 to February 2018. Some factors resulted in missing samples, which were, death of the patient, surgery during the time period of sampling, technical errors, vascular conditions not allowing to draw blood and transfer to another hospital unit (**Supplementary Figure 1**). Demographics and clinical characteristics of enrolled patients are shown in **Supplementary Table 1**. The study has been approved by the local Ethics Committee (file number: S-200/2017) according to the latest Declaration of Helsinki. All patients or their legal designees gave written informed consent before sample collection. The criteria for inclusion were based on the Sepsis-3 definition and clinical criteria (36). 19 samples of healthy controls were obtained from volunteers (file number: S-305/2010) without immunosuppression, autoimmune or infectious diseases. Upon the onset of sepsis, heparinized blood and serum tubes were drawn once daily for seven days. Within blood samples, leukocytes were stained directly and analyzed by flow cytometry. Serum sample were stored at -80°C for further investigation.

### Patient Data Collection

Parallel to collecting blood samples to investigate ASC-speck formation, clinical and laboratory data were recorded. Disease severity was determined using acute physiology and chronic health evaluation II (APACHE II), sequential organ failure assessment (SOFA) and simplified acute physiology score II (SAPS II) scores. The white blood cell count (WBC) and the concentration of c-reactive protein (CRP), lactate dehydrogenase (LDH) and procalcitonin (PCT) were also measured. Patient survival was followed up for 90 days.

### Determination of Intracellular ASC-Specks

Intracellular ASC-speck formation *in vivo* was determined by flow cytometry on fresh whole blood samples according to previous studies (30–32). The gating strategy is shown in **Supplementary Figure 2**. The following antibodies were used: CD3-PerCP, CD19-PerCP, CD56-PerCP, CD66b-PerCP, ASC-PE, and HLA-DR PE-Cy7 are from BioLegend (San Diego, CA); CD14 APC-Cy7 and CD16 Krome Orange are from BD Bioscience (Heidelberg, Germany) and Beckman Coulter (Krefeld, Germany), respectively. Samples of sepsis patients and healthy controls were processed in exactly the same way. In brief, 300 µl of fresh whole blood was incubated with the antibodies (CD3, CD19, CD56, CD66b, HLA-DR, CD14, and CD16) for 15 min at room temperature (RT) in the dark. Erythrocytes were lysed using FACS lysing solution (BD Biosciences) and leukocytes were fixed simultaneously. Cells were washed and permeabilized using 0.1% saponin followed by incubation with anti-ASC monoclonal antibody for 40 min at 4°C in the dark. After staining, cells were washed and flow cytometry was performed on a Gallios Flow Cytometer (Beckman Coulter). Counting beads (Molecular Probes Invitrogen, Paisley, UK) were added into each sample before acquisition to study absolute numbers of different leukocytes subsets. Flowjo software (Version 10.0.7 for windows) was used for flow cytometric analysis.

### Serum Cytokines and Chemokine Levels

Serum samples of patients on day 1, day 3, day 5, day 6, and day 7 were investigated. Levels of different cytokines including IL-1β, IL-18, IL-6, tumor necrosis factor-α (TNF-α), monocyte chemoattractant protein-1 (MCP-1), interferon-γ (IFN-γ), IL-12p70, IL-8, IL-10, thymic stromal lymphopoietin (TSLP), IL-1α, granulocyte macrophage colony stimulating factor (GM-CSF), IFN-α2, IL-23, IL-12p40, IL-15, IL-11, IL-27, IL-33, and IL-17A were determined using the legendplex multi-analyte flow assay kit human inflammation panel and human cytokine panel 2 (BioLegend). Assays were performed according to the manufacturer’s instructions.

### Statistical Analysis

Statistical analysis was undertaken using legendplex data analysis software (version 8.0), prism (version 5.0, graph pad software, La Jolla, CA, USA) and SPSS (version 21.0; SPSS Inc., Chicago, IL, USA). Comparisons of demographics and clinical characteristics between different groups were assessed using independent samples t tests, Mann-Whitney U tests, chi-square tests, or Fisher’s exact tests, as appropriate. Dot plots and bar-graph data are represented as median or mean ± standard error of the mean (SEM). The difference of ASC-speck^+^ cells between healthy controls and patients was determined by Mann-Whitney U test. Multiple comparisons among different time points of patient samples were analyzed by Kruskal–Wallis test. Correlations were assessed by Pearson coefficient. Receiver operating characteristic (ROC) curve analysis and the area under the ROC curve (AUROC) was applied to identify the optimal cutoff value. Kaplan-Meier analysis was used to calculate overall survival and the log-rank test was performed to assess the differences between survival curves. In univariate analysis, variables associated with mortality were assessed using the log-rank test. The predictors with a p value of <0.05 from the univariate analyses were included in multivariate analysis using a Cox proportional hazards model. Independent predictors of mortality were identified in the multivariate analysis and hazard ratios were computed for significant risk factors. For complete analysis, a two-tailed p value of less than 0.05 was considered as statistically significant.

## Results

### ASC-Speck Formation Among Blood Leukocytes of Sepsis Patients

**Figure 1:**
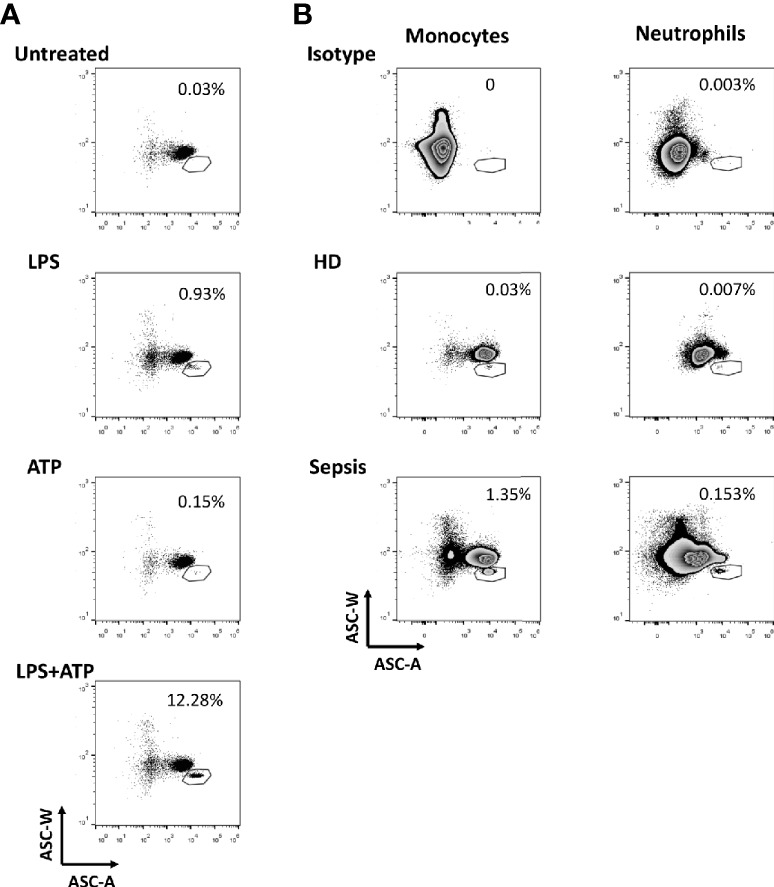
Formation of ASC-speck among leukocytes. **(A)** Detection of ASC-speck formation in peripheral blood mononuclear cells (PBMCs) of healthy donors after stimulation. Displayed are gated monocytes from gradient purified PBMCs (gating strategy is shown in **Supplementary Figure 2A**) cultured under different conditions including stimulation with lipopolysaccharides (LPS), with adenosine triphosphate (ATP) and with LPS + ATP. Gates were placed to include cells with a relatively low ASC-W: ASC-A profile (low W:A) that represent ASC-speck^+^ cells. Numbers shown next to the gates are the percentages of the ASC-speck^+^ cells from the HLA-DR^+^ monocyte population. Here, we confirmed that this flow cytometry assay allows the quantification of ASC speck-positive cells in samples. **(B)** Detection of ASC-speck formation in whole blood from sepsis patients and healthy donors. Displayed are gated monocytes and neutrophils from whole blood (gating strategy is shown in **Supplementary Figure 2B**). ASC-speck^+^ cells were defined by relatively low W:A in monocytes and neutrophils. Numbers shown next to the gates are the percentages of the ASC-speck^+^ cells from the HLA-DR^+^ monocyte and neutrophil population.

After inflammasome activation, ASC assembles to a protein complex, termed “speck”. The intracytoplasmic redistribution of ASC from a diffuse state into a single speck can be detected by a decreased width of the pulse of emitted fluorescence after staining with ASC-specific antibodies. Initially, we validated this flow cytometric assay (30–32) by using peripheral blood mononuclear cells (PBMCs) stimulated with lipopolysaccharide (LPS) and/or adenosine triphosphate (ATP) (**Supplementary Figure 2A** and **Figure 1A**). As expected, following cell stimulation with LPS and ATP, we observed a marked increase of inflammasome formation in monocytes. When studying patient samples and healthy controls (**Supplementary Figures 2B, C** and **Figure 1B**) we detected a far higher frequency of ASC-speck formation in monocytes and neutrophils in a representative sepsis patient. Further analysis showed that most of the ASC-speck^+^ monocytes belong to the population of CD14^++^CD16^-^ classical monocytes (**Supplementary Figure 3**). The flow cytometric gating strategy and respective isotype controls are shown in **Supplementary Figure 2** and the patient characteristics are provided in **Supplementary Table 1**.

### ASC-Speck^+^ Neutrophils and Monocytes Are Increased at Different Time Points in Sepsis Patients

**Figure 2:**
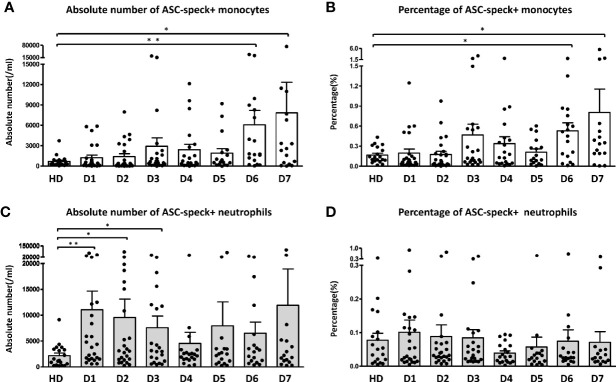
Dynamic change of ASC-speck^+^ cells in different leukocyte subsets. Both absolute number **(A)** and percentage **(B)** of ASC-speck^+^ monocytes increased significantly on day 6 and 7. The absolute number **(C)** of ASC-speck^+^ neutrophils increased during the first three days. However, no significant change of the percentage **(D)** of ASC-specks in neutrophils within seven days has been observed. The difference between healthy donors and patients was determined by Mann-Whitney test. *p < 0.05, **p < 0.01. Healthy donors n = 19; Sepsis patients day1 n = 24, day 2 n = 24, day 3 n = 22, day 4 n = 20, day5 n = 17, day 6 n = 18, day 7 n = 16. Dot plots and bar graph data are represented as mean ± SEM.

During the first week of sepsis we observed dynamic changes in the frequency of ASC-speck^+^ leukocytes in sepsis patients. Both the absolute number (**Figure 2A**) and percentage (**Figure 2B**) of ASC-speck^+^ monocytes were found increased on day 6 and day 7 compared to healthy donors. An increased absolute number of ASC-speck^+^ neutrophils was only observed on day 1 (**Figure 2C**). Over time we did not observe significant changes in the percentage of ASC-speck^+^ neutrophils (**Figure 2D**).

### Dynamic Change of Downstream Cytokines and Clinical Parameters

**Figure 3:**
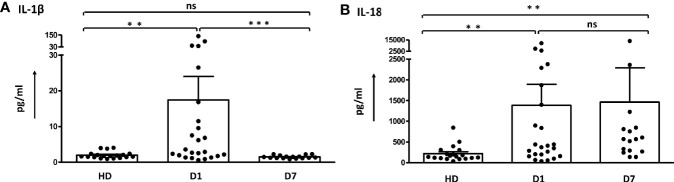
Dynamic change of serum levels of IL-1β and IL-18 in sepsis patients. **(A)** The concentration of IL-1β in sepsis patients was higher than in healthy donors on day 1 and decreased on day 7. **(B)** Values for IL-18 detected in sepsis patients remained increased. The difference was determined by Mann-Whitney test. **p < 0.01, ***p< 0.001. ns, not significant. Healthy donors n = 19; Sepsis patients day1 n = 24, day 7 n = 16. Dot plots and bar graph data are represented as mean ± SEM.

IL-1β and IL-18, the downstream cytokines secreted upon inflammasome activation, were determined in serum samples to further confirm the role of ASC-speck^+^ leukocytes in sepsis patients (**Figure 3** and **Supplementary table 2**). The concentration of IL-1β and IL-18 were found increased on day 1 compared to healthy controls. IL-1β values decreased back to baseline levels on day 7, whereas increased levels of IL-18 were maintained in the serum during the first week of sepsis. Other inflammatory cytokines were found elevated on day 1 and decreased thereafter (**Supplementary Figure 4** and **Supplementary Table 2**). The differences of cytokines between survivors and non-survivors were not significant (**Supplementary Table 3**).

The dynamic changes of LDH, WBC and PCT are depicted in **Supplementary Figure 5A**. There was a positive correlation between the frequency of ASC-speck^+^ monocytes (days 5–7) and the concentration of LDH and the WBC on days 5–7 (**Supplementary Figures 5B–D**).

### Low Absolute Number of ASC-Speck^+^ Blood Monocytes in Sepsis Non-Survivors

**Figure 4:**
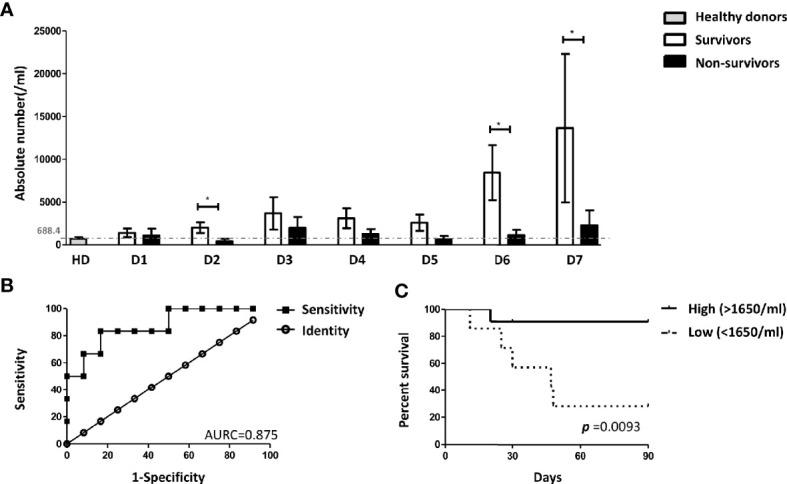
The absolute number of ASC-speck^+^ monocytes predicted the 90-days survival of sepsis patients. **(A)** Patients with a higher absolute number of ASC-specks on day 6 have a favorable outcome within 90 days *p < 0.05. Healthy donors n = 19; sepsis patients (survivors) day 1 n = 17, day 2 n = 17, day 3 n = 16, day 4 n = 14, day 5 n = 11, day 6 n = 12, day 7 n = 10; sepsis patients (non-survivors) day 1 n = 7, day 2 n = n = 7, day 3 n = 6, day 4 n = 6, day 5 n = 6, day 6 n = 6, day 7 n = 6. **(B)** Receiver operating characteristic (ROC) curve analysis on day 6 showed the AUROC for the absolute number of ASC-speck^+^ monocytes predicting survival was 0.875 (p = 0.011, 95% confidence interval (CI): 0.699–1.051) and the best the cutoff value was 1650 cells/ml with the highest sensitivity (83.33%) and specificity (83.33%). **(C)** Kaplan-Meier analysis revealed that patients with higher levels of ASC-speck positive monocytes (>1650 cells/ml) on day 6 have better survival [p = 0.0079, hazard ratio (HR) = 10.23, 95% CI: 1.840–56.94]. The patient cohort included 11 survivors and 7 non-survivors.

Next, we asked whether 90-day sepsis survivors and non-survivors differ in their frequency of ASC-speck^+^ blood leukocytes. As shown in **Figure 4A** this analysis revealed that sepsis non-survivors have significantly lower absolute number of ASC-speck^+^ monocytes on day 6 after the onset of sepsis. A further analysis was undertaken using ROC curves demonstrating that the absolute number of ASC-speck^+^ monocytes on day 6 discriminated with highest accuracy between survivors and non-survivors (**Figure 4B**). The AUROC for predicting survival was 0.875 [95% confidence interval (CI): 0.699–1.051]. The cut-off level of ASC-speck positive monocytes was 1,650 cells/ml with a specificity 83.33% (95% CI: 51.59–97.91%) and a sensitivity 83.33% (95% CI: 35.88–99.58%). Correspondingly, Kaplan-Meier curves were calculated to investigate whether the absolute number of ASC-speck^+^ monocytes serves as a putative biomarker in sepsis. These studies revealed that patients with lower absolute number of ASC-speck^+^ monocytes (<1,650 cells/ml) on day 6 showed an inferior survival [p = 0.0079, hazard ratio (HR) = 10.23, 95% CI: 1.840–56.94] (**Figure 4C**). Demographics and clinical characteristics of patients with high (>1,650 cells/ml) and low (<1,650 cells/ml) absolute number of ASC-speck^+^ monocytes are shown in **Supplementary Table 4**. The cut-off values for other parameters were also calculated by ROC curve analysis (**Supplementary Table 5**). Finally, the univariate and multivariate analysis (**Table 1**) showed that absolute number of ASC-speck^+^ monocytes is an independent predictor of 90-day mortality in sepsis patients (p = 0.035, HR = 10.178, 95% CI: 1.180–87.786).

**Table 1:** Univariable and multivariate analysis for 90-day mortality (n=18).

	Univariable analysis				Multivariable analysis		
Variables	P value	HR	95% CI		P value	HR	95% CI
mASC (AB) (>1650 vs.<1650 /ml)	**0.008**	10.23	1.840-56.94		**0.035** ^a^	10.178	1.180-87.786
mASC (%) (>0.1994 vs. <0.1994 %)	**0.039**	6.446	1.095-37.97		0.637^b^	–	–
SOFA (>6.5 vs. <6.5)	0.207	10.20	0.276-376.6		–	–	–
SAPS II (>70.5 vs. <70.5)	0.115	0.259	0.048-1.387		–	–	–
APACHE II (>34.5 vs.<34.5)	0.079	0.170	0.023-1.227		–	–	–
Weight (>66.00 vs. <66.00 kg)	**0.010**	12.80	1.856-88.3		0.127 ^b^	–	–
Age (>78 vs. <78 years)	**0.017**	0.065	0.007-0.616		0.383 ^b^	–	–
Gender (Male vs. Female)	0.847	1.171	0.235-5.842		–	–	–
Grade (Sepsis vs. Sepsis shock)	0.965	1.050	0.118-9.335		–	–	–
Lung (Yes vs. no)	0.226	3.482	0.462-26.22		–	–	–
Abdomen (Yes vs. no)	0.618	1.605	0.250-10.32		–	–	–
Gram-positive (Yes vs.no)	0.104	0.264	0.053-1.315				
Gram-negative (Yes vs.no)	0.054	5.579	0.972-32.02				
Fungal (Yes vs. no)	0.521	0.533	0.078-3.644				
Mixed (Yes vs. no)	0.692	0.720	0.142-3.652				
IFN-γ (>3.180 vs. <3.180 pg/ml)	0.087	0.225	0.041-1.243				
IL-10 (>222.7 vs. <222.7 pg/ml)	0.069	0.089	0.007-1.212				
IL-12p70 (>1.060 vs. <1.060 pg/ml)	0.226	0.287	0.038-2.163				
IL-18 (>331.2 vs.<331.2 pg/ml)	0.064	0.219	0.044-1.095				
IL-1β (>6.840 vs. <6.840 pg/ml)	0.239	0.378	0.075-1.907				
IL-6 (>53952 vs. <53952 pg/ml)	0.069	0.089	0.007-1.212				
IL-8 (>99.80 vs. <99.80 pg/ml)	0.226	0.287	0.038-2.163				
MCP-1 (>3179 vs. <3179 pg/ml)	0.604	0.649	0.126-3.336				
TNFα (>1.085 vs. <1.085 pg/ml)	0.226	0.287	0.0381-2.163				

Univariable analysis was performed by log-rank test. Predictors with p<0.05 from the univariate analyses were included in multivariate analysis. Multivariable analysis was performed using multivariable Cox proportional hazards regression.

mASC (absolute number), absolute number of ASC speck positive monocytes; mASC (%), the percentage of ASC specks in monocytes. CI, confidence interval; bold values for p<0.05.

mASC (absolute number) and mASC (%) values are from day 6 after onset of sepsis; values of all the other variables are from day 1 after onset of sepsis. ^a^,variables in the equation; ^b^,variables not in the equation.

In addition, we analyzed the expression of HLA-DR on CD14+ monocytes (**Supplementary Figure 6**). Gating was done as shown in **Supplementary Figure 6A**. When compared with healthy donors, the HLA-DR expression of monocytes obtained from sepsis patients was significantly reduced (**Supplementary Figure 6B**). Although not statistically significant monocyte HLA-DR expression was higher in survivors than in non-survivors (**Supplementary Figures 6C–E**).

## Discussion

Patients with prolonged immune suppression after sepsis frequently die from recurrent or hospital acquired infections (6–10, 12, 13). Reliable biomarkers identifying patients at risk of late death from sepsis are missing (15).

In the study presented here we identified a reduced *in vivo* inflammasome activation of blood monocytes as a prognostic factor of late death from sepsis. Recognition and killing of invading organisms by monocytes are important mechanisms of a protective innate inflammatory immune response. Equally important is the role of monocytes in presenting antigens by means of HLA molecules. Multiple studies have demonstrated clearly that following sepsis, monocytes have a diminished capacity for both of these responses. Specifically, they secrete fewer cytokines when stimulated (37) and down-regulate expression of HLA receptors (38, 39). This down-regulation of monocyte function generally predicts increased risk of secondary infection and poor prognosis (40).

Inflammasome activation is of central importance for the immune defense against infections and its role in sepsis pathophysiology has been highlighted by several studies recently (16–21). Among the different inflammasomes, NLRP3 and absent in melanoma 2 (AIM2) contain the adaptor molecule ASC. Inflammasomes such as the NLR family PYD containing 1 (NLRP1) and the NLR Family Caspase Recruitment Domain (CARD) Containing 4 (NLRC4) assemble in the absence of ASC, however, the presence of ASC enhances their activation and caspase-1 function (41, 42). Among the different inflammasomes NLRP3 is activated in response to the widest array of stimuli, including components from bacterial, viral, and fungal pathogens as well as endogenous molecules, for example uric acid crystals and stress signals, such as ATP and ion fluxes (43–50) converge into a common activating signal, which is the potassium efflux (51–54).

To monitor the systemic inflammatory responses in sepsis we studied *in vivo* inflammasome activation identified by ASC-speck formation in blood leukocytes during the first week of sepsis. Both neutrophils and monocytes are important cell subsets capable of inflammasome activation after stimulation. Being the most abundant leukocytes in the blood, neutrophils are the first line defense in protecting the host from infection or tissue damage (55). In this initial phase of sepsis bacterial derived pathogen-associated molecular patterns (PAMPs) may directly stimulate cells through their pathogen recognition receptors (PRRs). Activation of inflammasomes, release of cytokines, phagocytosis, degranulation and neutrophil extracellular trap formation are considered instrumental for this wave of host defense (4, 52–54). During the first 3 days of sepsis, neutrophils showed a significantly increased formation of ASC-specks. A short-lived rise of multiple inflammatory serum parameters (IL-1ß, TNF-α, IL-6, IL-8, MCP-1, PCT and CRP) accompanied this early response. Correspondingly, PCT values indicating bacteremia peaked on day 1 and declined thereafter, showing that infection in our patients was rapidly controlled (56).

Unlike other cytokines, IL-18 and IL-1ß are synthesized as precursor proteins and require enzymatic cleavage by caspase-1 to generate soluble, biologically active cytokines. In general, IL-1ß and IL-18 are produced and regulated differentially, and serum levels may not run in parallel (**Figure 3** and **Supplementary Figure 4A**). Secretion of mature IL-1ß requires cell activation, is usually short lived and largely inflammasome dependent (57, 58). In contrast, IL-18 secretion is partially inflammasome-independent, is increased and sustained after stimulation but also constitutively expressed in neutrophils, monocytes, intestinal epithelial cells and endocrine tissues, such as the adrenal gland (42, 58–61).

The key finding of this study was an increase in the absolute number and the percentage of ASC-speck^+^ monocytes on day 6 and 7 of sepsis. A detailed analysis then clearly revealed that patients with lower absolute numbers of ASC-speck^+^ monocytes on day 6 showed an inferior 90-day survival.

The stimulus of inflammasome activation in monocytes on day 6 is not clear. Multiple molecular and cellular signaling events, including ionic flux, mitochondrial dysfunction, the production of reactive oxygen species (ROS), and lysosomal damage, have been shown to activate the NLRP3 inflammasomes. We found LDH values to be still upregulated. LDH levels are directly related to tissue injury, serving as a marker of necrosis and hypoxia; collectively called danger-associated molecular patterns (DAMPs) (62, 63). Similar to PAMPs also DAMPs are recognized *via* PRR and may have caused inflammasome activation of immune cells on day 6 of sepsis. Interestingly, we found a correlation of serum LDH levels and ASC-speck^+^ monocytes on days 5–7 of sepsis (**Supplemental Figure 5**).

The role of inflammasome activation in sepsis has been addressed recently. In a study by Giamarellos-Bourboulis et al., reduced *in vitro* NLRP3 inflammasome activation and IL-1ß production of monocytes stimulated with monosodium urate crystal was observed in sepsis patients compared to healthy controls (64). A study by Weighardt et al. found higher IL-1ß production of ex vivo stimulated monocytes in sepsis survivors compared to non-survivors (65). Similar results were obtained by Martinez-Garcia et al. (20); interestingly, these authors also addressed a molecular mechanism responsible for the hyporesponsiveness of monocytes. The authors showed that activation of cells *via* the purinergic ATP receptor P2X7 results in mitochondrial dysfunction causing inflammasome inhibition and immunosuppression of monocytes (20). Therefore, ATP release with P2X7 receptor activation as a consequence of different treatments or complications already prior to sepsis can be seen as one possibility to disarm monocyte inflammasomes causing immunoparalysis and late death from sepsis (20).

Interestingly, low level *in vivo* formation of ASC-specks in monocytes during early sepsis (day 1 to 5) was found in all patients, which happened despite the obvious PAMP-induced neutrophil activation evidenced by increased numbers of ASC-speck^+^ neutrophils and increased cytokine serum levels. A reduced inflammasome activation of monocytes during early sepsis was previously found in studies on *in vitro* activated monocytes (64, 65) and appears to be a general phenomenon. Whether this dysfunction of monocytes resulted from the initial PAMP-induced and granulocyte-dominated inflammatory response, is not known. Subsequent immunorestoration with successful induction of ASC-speck^+^ monocytes (>1,650 cells/ml) on day 6 of sepsis—possibly stimulated by DAMPs—appeared highly beneficial in terms of sepsis survival. Thus, some patients appear capable of escaping the hypo-inflammatory phase while others with lower amount of ASC-speck^+^ monocytes on day 6 may still be locked in a state of “immunoparalysis” associated with inferior survival. Enhanced inflammasome activation of monocytes is associated with increased rates of cell death by pyroptosis and indicative of a higher monocyte turnover (66). Ultimately, it is currently unknown whether ASC-speck^+^ monocytes directly save patients from post sepsis mortality or whether ASC-speck^+^ monocytes are indicative for a competent immune system, with a higher monocyte turnover, ready to fight off invading pathogens.

The activation of inflammasome involves two distinct steps. Firstly, a transcription factor nuclear factor-κB (NF-κB)-dependent manner, such as the binding of LPS to its receptor Toll-like Receptor (TLR)4, induces elevated expression of pro-IL-1ß. Secondly, P2X7 receptor-mediated K+ efflux induces inflammasome assembly, as well as the subsequent maturation and release of IL-1ß (52, 67, 68). ASC-speck^+^ monocyte numbers increased significantly during the later phase of sepsis (day 6 and day 7), when, however, serum IL-1ß levels were back to normal. This may appear as a contradiction at first; however, detection of ASC-speck in blood leukocytes represents only one source of the total IL-1ß producing cells contributing to IL-1ß serum levels, and the IL-1ß assay detected not only the inflammasome-dependent mature form of IL-1ß but also the unprocessed pro-IL-1ß, which is released upon cell lysis caused by multiple pathways including pyroptosis. In addition, production of IL-1ß depends on a two-step process regulated at the mRNA- in addition to the inflammasome level.

Monocytes are not only essential elements in innate immune responses, they also express MHC-class II and are regarded to play an important role in orchestrating adaptive immunity. Previous studies reported that persisting monocyte deactivation, characterized by a decrease in HLA-DR expression from the initial period, was associated with higher mortality and the recovery of function of monocytes was observed in survivors within 10 days (11, 38, 69–72). As previously described, we noted that HLA-DR expression of monocytes in sepsis patients was significantly reduced (70, 71). There was a clear trend that the difference of HLA-DR expression on day 6 compared to the first 4 days was higher in patients surviving sepsis. These differences did not reach statistical significance in our cohort (**Supplementary Figure 6**). In addition, for our patients we did not observe a statistically significant association of survival and different clinical scores (APACHE II, SOFA, or SAPS II).

Studies with murine sepsis models showed the different roles of inflammasomes. Wegiel et al. reported that the NLRP3 inflammasome is relevant for bacterial clearance in mice (73). While Lee et al. found that genetic deficiency of NLRP3 inhibited inflammatory responses and permitted enhanced survival of septic mice (17). In line with the notion that overwhelming inflammation is detrimental Dolinay et al. reported that caspase-1–dependent inflammatory responses involving the production and activation of IL-18 may play a role in the propagation of acute respiratory distress syndrome (74). The focus of many studies is the overwhelming inflammatory response at the beginning of sepsis leading to immunoparalysis and death. We instead monitored the patients for one week and identified a marker correlating with immune recovery and sepsis survival.

## Conclusion

Inflammasome research and monocyte function are of particular interest in sepsis (18, 20, 21). In the study presented here, *in vivo* inflammasome activation was identified by ASC-speck formation in blood leukocytes during the first week of sepsis. We found that both neutrophils and monocytes are important cell subsets capable of inflammasome activation in sepsis. Statistical analysis demonstrated that the frequency of ASC-speck^+^ monocytes on day 6 after onset of sepsis may serve as a marker for immune monitoring and identifying patients at risk of death.

Although it remains unclear how ASC-speck^+^ monocytes rescue patients from death of sepsis, our present results provide a novel link between *in vivo* inflammasome activation in monocytes, immune competence and sepsis survival. The inflammasome has well-known pleiotropic and potentially “double-edged sword” effects in sepsis as a regulator of inflammation and as an integral part of the immune response. Future studies are required to describe the exact role of the rapidly performed flow cytometric assay to identify ASC-speck^+^ monocytes in making clinical decisions in sepsis.

## Data Availability Statement

The original contributions presented in the study are included in the article/**Supplementary Material**. Further inquiries can be directed to the corresponding author.

## Ethics Statement

The studies involving human participants were reviewed and approved by Ethics committee of the University of Heidelberg, described in the *Methods* in more detail. The patients/participants provided their written informed consent to participate in this study.

## Author Contributions

JC, SO, FA, FF, CR, and SM performed the experiments. JC, SO, TB, FU, CN, MW, CM, and KS designed the project and interpreted the data. JC, SO, and KS. wrote the manuscript. TB, FU, CN, MW, and CM critically read the manuscript and facilitated sample collection. KS has final responsibility for decision to submit publication. All authors contributed to the article and approved the submitted version.

## Conflict of Interest

The authors declare that the research was conducted in the absence of any commercial or financial relationships that could be construed as a potential conflict of interest.
