# Supplementary material for: Detection of In Vivo Inflammasome Activation for Predicting Sepsis Mortality
Source: Front Immunol. 2021 Feb 4;11:613745. doi: 10.3389/fimmu.2020.613745 (PMC7889521; doi:10.3389/fimmu.2020.613745)
Supplement: Supplementary file 1 [file DataSheet_1.docx]

**Supplementary Figure 1. Flowchart of sample collection.**

* Reduced sample number due to death of patients or missing blood samples.

In total, 25 patients with sepsis or septic shock were included and 141 out of 175 possible samples were collected. A number of samples were missing for different reasons: death (1 patient on day 2), bad vascular condition resulting in failure of drawing blood (in total 6 missing samples on different days), surgery during collection period (in total 10 missing samples on different days), recovery (1 patient on day 6) and technical errors (11 samples on different days).

**Supplementary Figure 2. Gating strategy. (A)** Gradient purified peripheral blood mononuclear cells (PBMCs) from healthy donors were treated with different conditions. The predominant population of paraformaldehyde (PFA) fixed cells viewed on a SSC-A vs. FSC-A dot plot were selected. Then SSC-W vs. SSC-H and FSC-W vs. FSC-H dot plots were used to exclude cell doublets. Afterwards, HLA-DR^+^ cells were gated. Finally, the HLA-DR^+^ cells were plotted by relative expression of CD14 and CD16. A gate was placed to include all monocyte subset. **(B)** Whole blood staining. Debris and clumps were excluded and lineage-specific marker CD3, CD19 and CD56 were used for the definition of the different lymphocyte subsets. Simultaneously, the identification of neutrophils was undertaken using CD66b. Then HLA-DR^+^ monocytes were gated by HLA-DR PE-Cy7, CD14-APC-Cy7 and CD16-Krome Orange. **(C)** Isotype control. Displayed are gated monocytes and neutrophils from whole blood (gating strategy is shown in Supplementary Figure 2B). ASC-speck^+^ cells were defined by relatively low W:A in monocytes and neutrophils. Numbers shown next to the gates are the percentages of the ASC-speck^+^ cells from the HLA-DR^+^ monocytes and neutrophils.

**Supplementary Figure 3. The formation of ASC-speck in different monocyte subsets.** **(A)** Representative flow cytometric analysis of monocyte subsets, based on CD14 and CD16 expression (CD14^++^CD16^-^ classical, CD14^+^CD16^+^ intermediate and CD14^low^CD16^+^ non-classical monocytes), then ASC-speck^+^ cells were gated in the three monocyte subsets respectively. **(B)** Stacked bar graphs show that the absolute number of ASC-speck^+^ classical, intermediate and non-classical monocytes in healthy donors (HD) and sepsis patients between day 1 (D1) and day 7 (D7). **(C)** Stacked bar graphs show that the proportion of ASC-speck^+^ classical, intermediate and non-classical monocytes in HD and sepsis patients from D1 to D7 (the total proportion in every column is 100%).

**Supplementary Figure 4. Changes of cytokine serum levels.** Dynamic changes of the inflammasome-dependent cytokines IL-1β and IL-18 (A) and other studied cytokines (B) are shown. Data are presented as mean ± standard error of the mean (SEM). Dunn's Multiple Comparison Test was used for testing the difference of the cytokine levels between Day 1 and other days. * p < 0.05, ** p < 0.01, *** p < 0.001. Sepsis patients day1 n=24, day 3 n=22, day5 n=17, day 6 n=18, day 7 n=16.

**Supplementary Figure 5. Correlation between ASC-specks and clinical parameters in sepsis patients. (A)** No significant change of LDH and WBC count has been observed within one week, Kruskal-Wallis test p>0.05. The level of PCT declined from Day 1 to Day 7 significantly, Kruskal-Wallis test p<0.0001. Data is represented as mean ± standard error of the mean (SEM). **(B)** Positive correlation was found between the percentage of ASC speck positive monocytes and the concentration of LDH on Day 5 and Day 7. Pearson’s correlation test: D 5 (r=0.5674, p=0.0175), D 6 (r=0.4638, p=0.0525), D 7 (r=0.5920, p=0.0175). **(C)** Positive correlation between the absolute number of ASC speck-expressing monocytes and LDH on Day 7. Pearson’s correlation test: D 5 (r=0.3993, p=0.1123), D 6 (r=0.4255, p=0.0791), D 7 (r=0.6334, p=0.0084). **(D)** Positive correlation between the absolute number of ASC speck positive monocytes and WBC count. The analysis of all correlations was undertaken using Pearson’s rank dot plot on Day 5, Day 6 and Day 7. Pearson’s correlation test: D 5 (r=0.6791, p=0.0027), D 6 (r=0.6101, p=0.0072), D 7 (r=0.6535, p=0.0060).

**Supplementary Figure 6. The expression of HLA-DR on monocytes. (A)** Displayed are gated monocytes from whole blood of sepsis patient: FSC-A, FSC-W, FSC-H, SSC-A, SSC-W and SSC-H were set to exclude debris and clumps. Afterwards, the lineage-specific markers CD3, CD19 and CD56 were used to exclude lymphocytes (T cells, B cells and NK cells), CD66b was used to exclude neutrophils. The CD14^+^ cells were gated as monocyte. **(B)** Monocyte HLA-DR levels, expressed as median of fluorescence intensity (MFI), between healthy donors and sepsis patient were compared. Healthy donors n=19; Sepsis patients day1 n=24, day 2 n=24, day 3 n=22, day 4 n=20, day5 n=17, day 6 n=18, day 7 n=16. Dot plots and bar graph data are represented as mean ± SEM. **(C)** Compared with Day 1, Day 2, Day 3 and D 4, the monocyte HLA-DR MFI on day 6 in 90-day survivors increased more than non-survivors. The statistical analysis showed no significant differences. Sepsis survivors n=12 (Δ6-1 and Δ6-2), n=11 (Δ6-3 and Δ6-4); Sepsis non-survivors n=6. **(D)** Compared with Day 1, Day 2, Day 3 and D 4, the monocyte HLA-DR MFI on day 6 in ASC-speck+ monocyte high group (>1650 cells/ml) increased more than high group. The statistical analysis showed no significant differences. High group n=11, low group n=7. Sepsis survivors n=11 (Δ6-1 and Δ6-2), n=10 (Δ6-3 and Δ6-4); Sepsis non-survivors n=7. Δ6-1 was defined as the value change in monocyte HLA-DR MFI on day 6 compared with that on day 1. The difference was determined by Mann-Whitney test. * p < 0.05; ** p < 0.01, *** p < 0.001.
