# Supplementary figures and images for: Detection of In Vivo Inflammasome Activation for Predicting Sepsis Mortality
Source: Front Immunol. 2021 Feb 4;11:613745. doi: 10.3389/fimmu.2020.613745 (PMC7889521; doi:10.3389/fimmu.2020.613745)

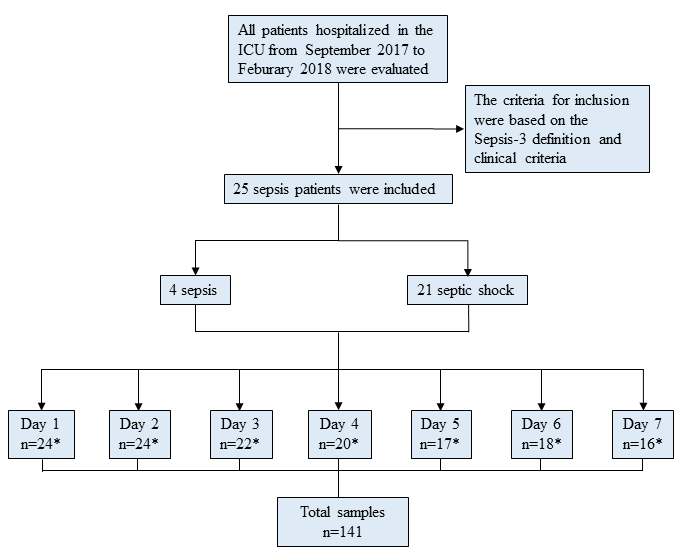

Supplement: Supplementary file 2 [file Image_1.tif]

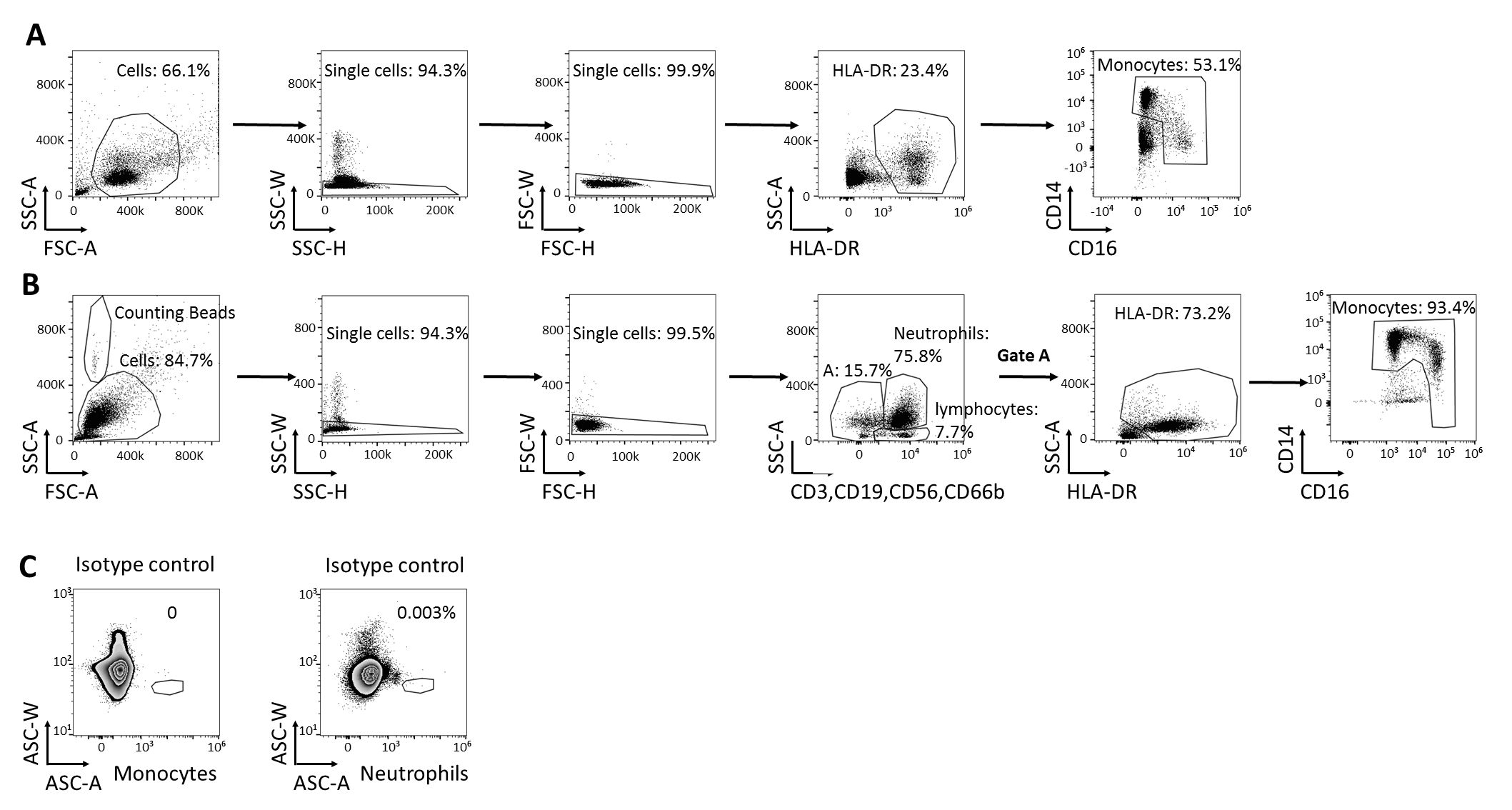

Supplement: Supplementary file 3 [file Image_2.tif]

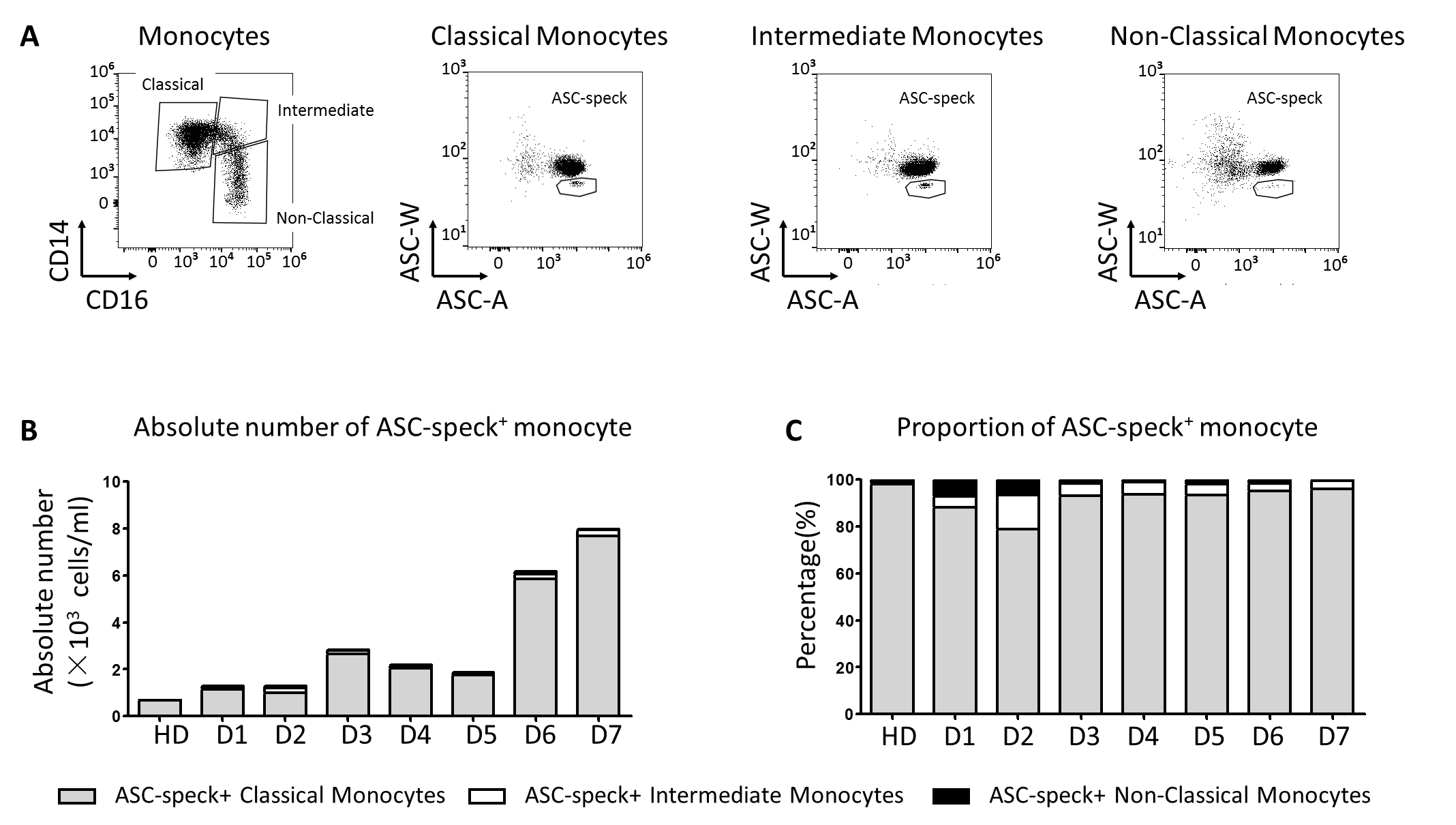

Supplement: Supplementary file 4 [file Image_3.tif]

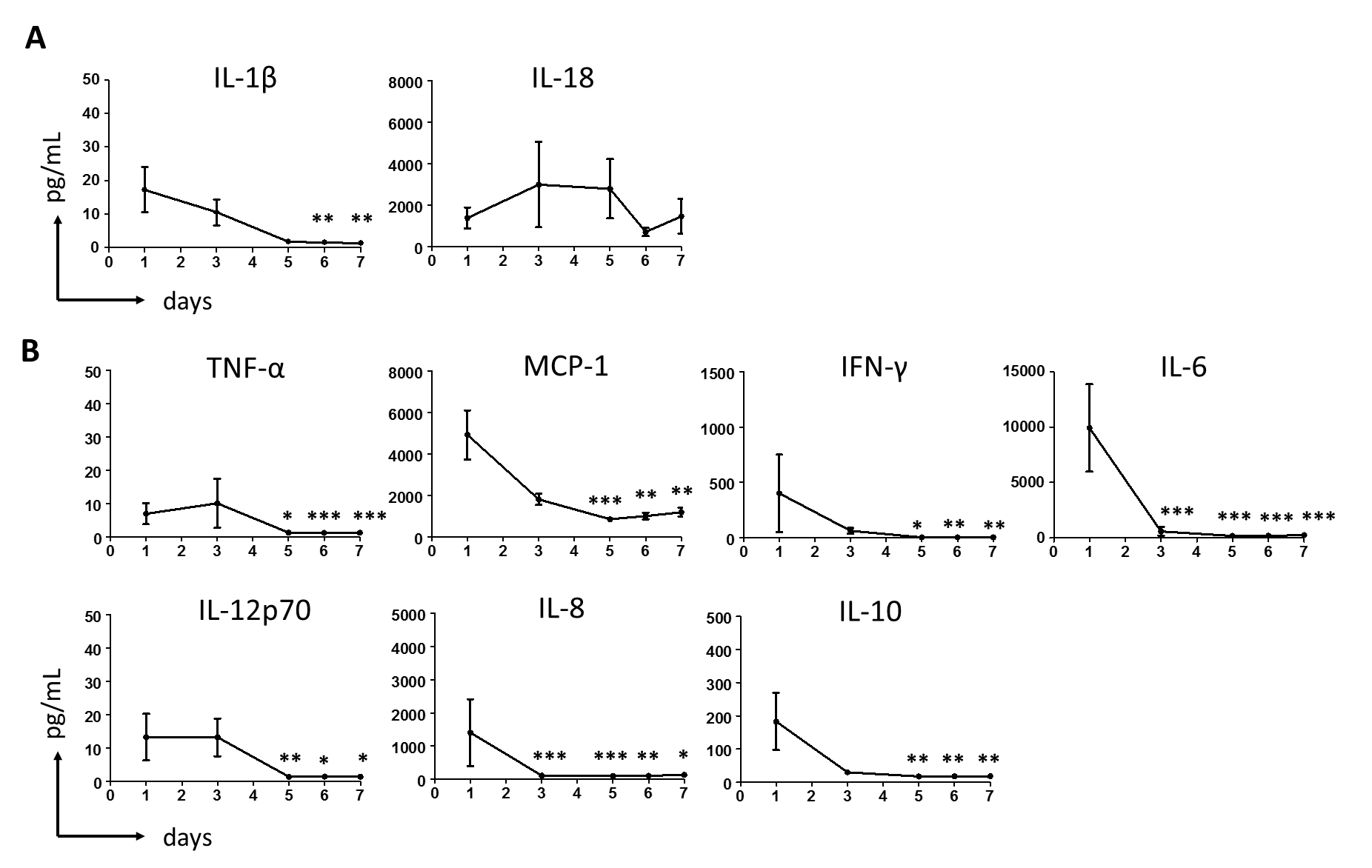

Supplement: Supplementary file 5 [file Image_4.tif]

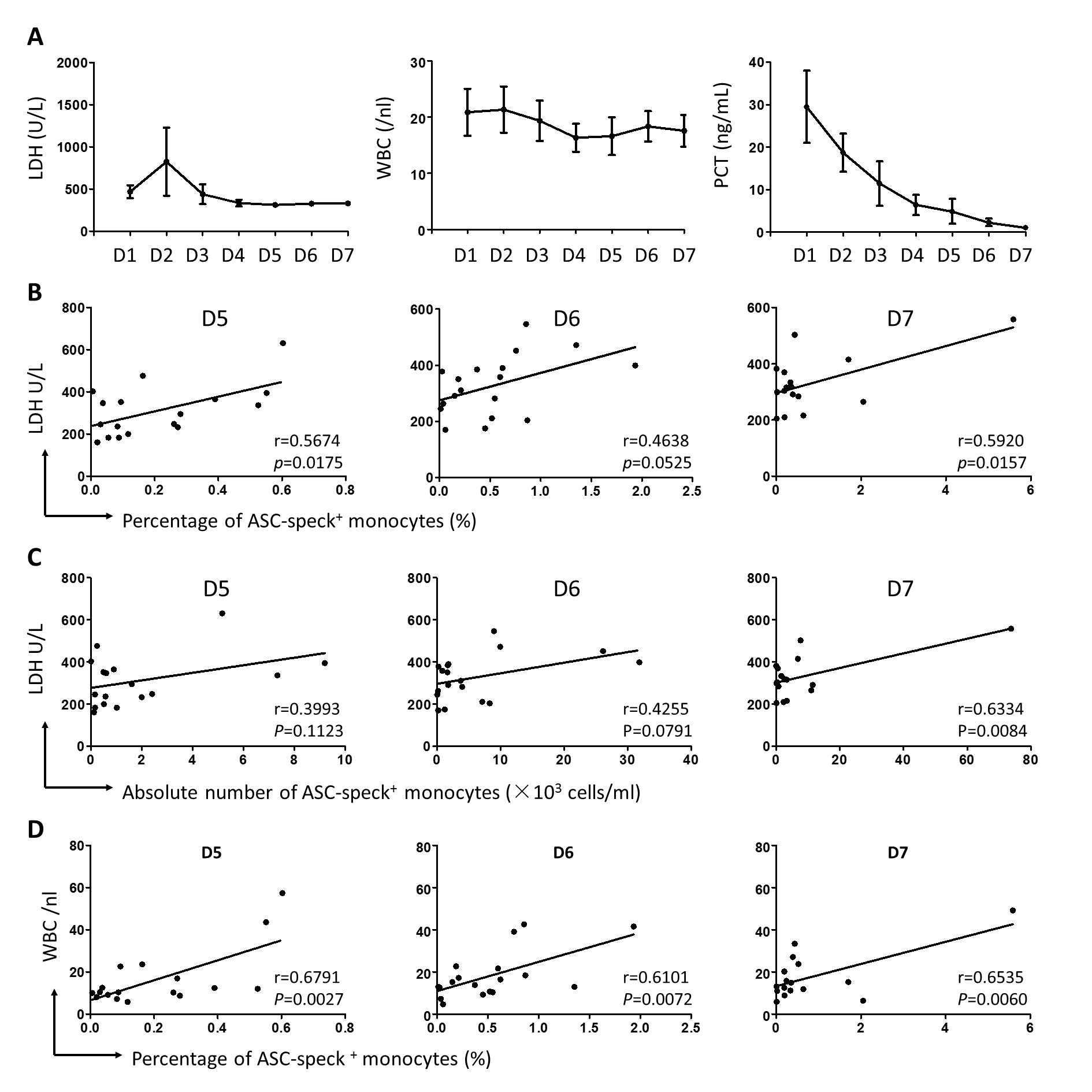

Supplement: Supplementary file 6 [file Image_5.tif]

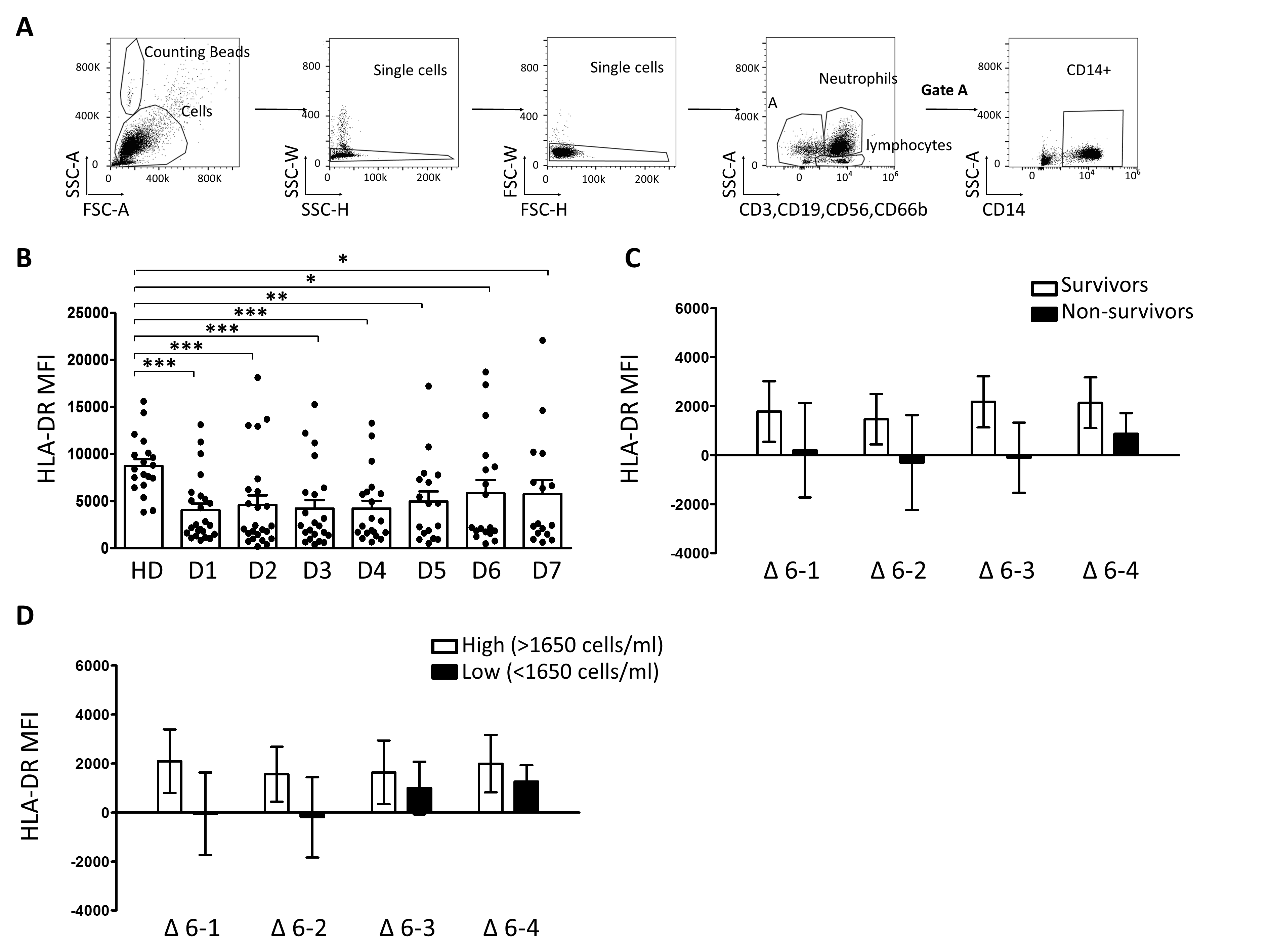

Supplement: Supplementary file 7 [file Image_6.tif]
