# Supplementary material for: Detection of In Vivo Inflammasome Activation for Predicting Sepsis Mortality
Source: Front Immunol. 2021 Feb 4;11:613745. doi: 10.3389/fimmu.2020.613745 (PMC7889521; doi:10.3389/fimmu.2020.613745)
Supplement: Supplementary file 8 [file Table_1.docx]

**Supplementary Table 1. Demographics and clinical characteristics of enrolled patients**

| Variables | All patients n=25 | 90-day survivors n=18 | 90-day non-survivors n=7 | p value |
| --- | --- | --- | --- | --- |
| Demographics |  |  |  |  |
| Age(years) | 64.00(59.00-77.00) | 63.50(56.50-75.00) | 71.00(61.50-80.50) | 0.205 |
| Weight(kg) | 78.00(67.00-90.00) | 82.50(75.00-91.50) | 65.00(59.00-81.50) | 0.179 |
| Height(cm) | 175.00(163.00-180.00) | 177.00(165.25-180.75) | 165.00(162.50-170.00) | 0.383 |
| Gender(male) | 14(56.00%) | 10(55.56%) | 4(57.14%) | 1.000 |
| Site of infection |  |  |  |  |
| Lung | 5(20.00%) | 4(22.22%) | 1(14.29%) | 1.000 |
| Abdomen | 19(76.00%) | 14(77.78%) | 5(71.43%) | 1.000 |
| Genitourinary tract | 3(12.00%) | 2(11.11%) | 1(14.29%) | 1.000 |
| Catheter | 1(4.00%) | 0(0%) | 1(14.29%) | 0.280 |
| Mediastinum | 2(8.00%) | 1(5.56%) | 1(14.29%) | 0.490 |
| Combined | 5(20.00%) | 3(16.67%) | 2(28.57%) | 0.597 |
| Culture positive | 24(96.00%) | 17(94.44%) | 7(100%) | 1.000 |
| Organism |  |  |  |  |
| Bacteria | 23(92.00%) | 16(88.89%) | 7(100%) | 1.000 |
| Gram-negative | 15(60.00%) | 9(50.00%) | 6(85.71%) | 0.179 |
| Gram-positive | 17(68.00%) | 14(77.78%) | 3(42.86%) | 0.156 |
| Fungal | 5(20.00%) | 3(16.67%) | 2(28.57%) | 0.597 |
| Viral | 0(0%) | 0(0%) | 0(0%) | - |
| Mixed | 14(56.00%) | 10(55.56%) | 4(57.14%) | 1.000 |
| Septic shock | 21(84.00%) | 15(83.33%) | 6(85.71%) | 1.000 |
| 14-day mortality | 2(8.00%) | - | 2(28.57%) | - |
| 28-day mortality | 4(16.00%) | - | 4(57.12%) | - |
| 90-day mortality | 7(28.00%) | - | 7(100%) | - |
| Clinical parameters |  |  |  |  |
| PCT(ng/ml) D1 or D2 | 8.65(2.49-43.30) | 9.015(3.54-39.72) | 2.36(0.76-44.77) | 0.348 |
| WBC(10^9^/L) D1 | 15.40(10.61-23.86) | 15.39(10.66-29.12) | 16.38(12.41-18.81) | 0.694 |
| WBC (10^9^/L) D2 | 14.13(9.75-25.31) | 14.90(11.16-27.84) | 9.85(9.18-19.08) | 0.263 |
| WBC (10^9^/L) D3 | 12.45(9.99-19.73) (n=24) | 12.97(10.43-17.93) | 11.175(9.68-24.78) (n=6) | 0.571 |
| WBC (10^9^/L) D4 | 11.46(9.77-16.69) (n=24) | 10.84(9.93-15.06) | 12.27(9.81-19.26) (n=6) | 0.714 |
| WBC (10^9^/L) D5 | 12.11(9.87-17.47) (n=24) | 12.18(10.17-15.93) | 10.40(8.69-19.60) (n=6) | 0.527 |
| WBC (10^9^/L) D6 | 13.87(10.57-18.45) (n=23) | 15.29(11.00-18.44) (n=17) | 11.70(8.08-19.52) (n=6) | 0.421 |
| WBC (10^9^/L) D7 | 13.13(11.00-19.45) (n=23) | 15.29(11.79-20.22) (n=17) | 11.13(7.55-12.17) (n=6) | 0.177 |
| CRP(mg/l)D1 | 182.30(91.70-258.40) | 186.65(145.68-277.23) | 91.70(59.8-204.05) | 0.107 |
| CRP(mg/l)D2 | 219.60(159.40-283.50) | 243.75(218.88-323.43) | 159.40(102.25-195.85) | **0.012** |
| CRP(mg/l)D3 | 197.10(154.75-266.33) (n=24) | 244.90(160.23-283.58) | 159.10(143.73-178.68) (n=6) | **0.017** |
| CRP(mg/l)D4 | 152.25(113.10-181.53) (n=24) | 153.95(107.10-186.18) | 150.45(134.65-154.93) (n=6) | 0.886 |
| CRP(mg/l)D5 | 100.40(79.83-138.73) (n=24) | 99.90(73.88-131.73) | 110.80(89.30-143.63) (n=6) | 0.738 |
| CRP(mg/l)D6 | 64.80(54.50-114.35) (n=23) | 64.80(55.30-112.70) (n=17) | 82.20(53.23-113.20) (n=6) | 0.550 |
| CRP(mg/l)D7 | 83.90(53.55-126.70) (n=23) | 83.90(56.50-127.20) (n=17) | 87.95(56.55-103.90) (n=6) | 0.916 |
| LDH(U/l)D1 | 312.00(244.00-468.00) | 329.00(267.50-500.25) | 284.00(232.00-333.50) | 0.468 |
| LDH(U/l)D2 | 292.00(234.00-459.00) | 286.50(216.50-372.25) | 438.00(254.50-1706.00) | 0.155 |
| LDH(U/l)D3 | 294.50(237.75-361.00) (n=24) | 294.50(243.25-343.00) | 295.00(235.25-423.75) (n=6) | 0.816 |
| LDH(U/l)D4 | 305.50(220.25-350.50) (n=24) | 326.50(224.75-344.75) | 245.00(218.00-346.25) (n=6) | 0.681 |
| LDH(U/l)D5 | 297.00(235.25-370.00) (n=24) | 318.00(241.00-380.00) | 247.00(238.50-326.00) (n=6) | 0.495 |
| LDH(U/l)D6 | 331.00(247.25-383.75) (n=23) | 374.00(243.75-392.25) (n=17) | 272.50(249.50-333.75) (n=6) | 0.213 |
| LDH(U/l)D7 | 317.50(269.75-406.75) (n=23) | 347.00(280.25-443.00) (n=17) | 291.50(269.75-302.75) (n=6) | 0.040 |
| Lactate(mg/dl)D1 | 19.90(13.30-38.50) | 19.85(14.33-28.85) | 25.60(15.65-60.20) | 0.506 |
| Lactate(mg/dl)D2 | 13.60(10.65-27.75) | 12.20(10.60-19.60) | 27.75(20.08-64.45) | 0.064 |
| Lactate(mg/dl)D3 | 11.05(9.55-17.35) (n=24) | 10.45(8.88-15.18) | 16.85(14.7-18.40) (n=6) | **0.036** |
| Lactate(mg/dl)D4 | 12.40(10.15-16.35) (n=24) | 12.40(9.90-14.90) | 13.20(10.63-17.65) (n=6) | 0.485 |
| Lactate(mg/dl)D5 | 10.75(9.45-16.48) (n=24) | 11.80(9.98-16.73) | 10.30(8.70-10.78) (n=6) | 0.417 |
| Lactate(mg/dl)D6 | 11.40(9.70-14.80) (n=23) | 11.40(9.40-15.20) (n=17) | 11.05(9.80-13.88) (n=6) | 0.786 |
| Lactate(mg/dl)D7 | 11.30(9.00-14.50) (n=23) | 11.30(9.40-14.55) (n=17) | 10.05(8.23-11.80) (n=6) | 0.938 |
| Scoring models |  |  |  |  |
| SOFA(D1) | 13.00(10.00-14.00) | 12.50(10.25-14.00) | 14.00(9.50-14.50) | 0.874 |
| SOFA(D2) | 11.00(9.00-15.00) | 10.50(9.00-12.75) | 16.00(7.50-17.50) | 0.466 |
| SOFA(D3) | 11.00(8.00-14.00) (n=24) | 10.50(8.00-13.00) | 13.50(7.25-16.75) (n=6) | 0.422 |
| SOFA(D4) | 9.00(6.75-12.25) (n=24) | 9.00(6.25-9.00) | 13.00(8.75-15.00) (n=6) | 0.146 |
| SOFA(D5) | 8.00(6.00-10.00) (n=24) | 8.00(6.00-9.75) | 11.50(6.75-14.75) (n=6) | 0.181 |
| SOFA(D6) | 8.00(4.50-10.00) (n=23) | 7.00(4.00-9.00) (n=17) | 11.00(6.00-13.75) (n=6) | 0.157 |
| SOFA(D7) | 6.00(3.50-8.00) (n=23) | 6.00(3.00-8.00) (n=17) | 11.50(5.50-15.25) (n=6) | 0.124 |
| SAPSⅡ(D1) | 66.00(52.00-78.00) | 64.50(51.25-70.00) | 76.00(63.00-81.50) | 0.416 |
| SAPSⅡ(D2) | 67.00(49.00-75.00) | 63.00(46.75-73.50) | 75.00(61.50-85.00) | 0.123 |
| SAPSⅡ(D3) | 62.50(47.50-67.75) (n=24) | 62.50(43.75-66.75) | 60.00(49.25-77.5) (n=6) | 0.326 |
| SAPSⅡ(D4) | 54.00(40.75-70.25) (n=24) | 53.50(39.50-59.50) | 71.00(49.00-81.75) (n=6) | 0.100 |
| SAPSⅡ(D5) | 49.50(37.75-60.75) (n=24) | 43.00(35.25-58.50) | 61.50(49.00-83.75) (n=6) | 0.056 |
| SAPSⅡ(D6) | 48.00(34.50-60.50) (n=23) | 45.00(34.00-59.00) (n=17) | 63.00(41.25-84.75) (n=6) | 0.101 |
| SAPSⅡ(D7) | 46.00(34.50-64.00) (n=23) | 44.00(31.00-48.00) (n=17) | 66.50(44.50-82.50) (n=6) | **0.037** |
| APACHEⅡ(D1) | 30.00(24.00-33.00) | 29.50(24.00-32.00) | 36.00(28.50-40.00) | 0.256 |
| APACHEⅡ(D2) | 31.00(25.00-34.00) | 29.00(20.50-33.75) | 32.00(30.00-35.00) | 0.508 |
| APACHEⅡ(D3) | 27.50(19.50-33.25) (n=24) | 27.50(18.50-32.50) | 26.50(21.00-34.25) (n=6) | 0.714 |
| APACHEⅡ(D4) | 24.50(19.00-28.5) (n=24) | 23.50(19.50-26.75) | 27.00(20.75-31.00) (n=6) | 0.277 |
| APACHEⅡ(D5) | 24.00(17.75-28.25) (n=24) | 22.00(17.25-27.50) | 29.00(23.50-30.00) (n=6) | 0.222 |
| APACHEⅡ(D6) | 22.00(20.00-28.50) (n=23) | 22.00(21.00-24.00) (n=17) | 29.00(17.50-30.75) (n=6) | 0.245 |
| APACHEⅡ(D7) | 23.00(15.50-29.00) (n=23) | 22.00(16.00-24.00) (n=17) | 30.50(18.75-34.00) (n=6) | 0.175 |

Variables were shown as frequencies (percentages) for categorical data and medians (interquartile ranges) for continuous data.

D1 mean the first day after onset of spesis, D2 means the second day after onset of sepsis……

The n value of non-survivors becomes 6 from Day 3, because 1 patient died on day 2.

The n value of survivors was 17 on Day 6, as samples of 2 patients were not available on day 6 and day 7.

Bold values for p<0.05

**Supplementary Table 2. Cytokines in serum samples of healthy donors and sepsis patients.**

| Variables | HD（n=19） | D1（n=24） | *p* value  HD vs. D1 | D3 (n=22) | *p* value  HD vs. D3 | D5 (n=17) | *p* value  HD vs. D5 | D6 (n=18) | *p* value  HD vs. D6 | D7 (n=16) | *p* value  HD vs. D7 |
| --- | --- | --- | --- | --- | --- | --- | --- | --- | --- | --- | --- |
| IL-1β | 1.99±0.22 | 17.41±6.63 | **0.0099** | 10.59±3.79 | 0.0868 | 1.92±0.19 | 1 | 1.69±0.19 | 0.1667 | 1.49±0.11 | 0.1082 |
| IL-18 | 221.02±44.04 | 1386.96±506.32 | **0.0106** | 940.97±164.61 | **<0.0001** | 1550.03±751 | **0.0011** | 713.69±200.71 | **0.0002** | 1464.62±829.39 | **0.0005** |
| TNFα | 1.46±0.09 | 6.96±3.18 | **0.0189** | 10.12±7.36 | 0.224 | 1.32±0.1 | 0.2223 | 1.19±0.11 | **0.0298** | 1.29±0.23 | **0.034** |
| MCP-1 | 738.77±102.56 | 4930.03±1183.58 | **<0.0001** | 1817.15±269.95 | **0.0039** | 860.1±109.51 | 0.3749 | 1013.64±165.44 | 0.086 | 1196.25±212.36 | 0.0945 |
| IFN-γ | 3.36±0.51 | 401.39±350.17 | **0.0024** | 63.3±25.48 | **0.013** | 3.68±0.69 | 0.8991 | 5.58±2.28 | 0.3539 | 5.03±1.53 | 0.7033 |
| IL-6 | 12.4±9.41 | 6777.33±3332 | **<0.0001** | 592.44±390.04 | **<0.0001** | 185.58±41.59 | **<0.0001** | 191.1±34.52 | **<0.0001** | 254.81±87.54 | **<0.0001** |
| IL-12p70 | 3.8±2.31 | 13.28±7 | **0.026** | 13.24±5.66 | **0.0484** | 1.36±0.11 | 0.612 | 1.44±0.13 | 0.704 | 1.38±0.11 | 0.631 |
| IL-8 | 20.69±5.18 | 391.56±79.53 | **<0.0001** | 112.82±18.7 | **<0.0001** | 96.65±19.48 | **<0.0001** | 105.59±14.74 | **<0.0001** | 132.15±20.6 | **<0.0001** |
| IL-10 | 3.51±1.63 | 183.1±86.29 | **<0.0001** | 29.74±7.34 | **<0.0001** | 17.49±5.88 | **<0.0001** | 17.94±5.25 | **<0.0001** | 18.03±6.21 | **<0.0001** |

HDs, healthy donors; D1, day 1 after the onset of sepsis; D3, day 3 after the onset of sepsis……

SEM, Standard Error of Mean.

Variables are described as mean±SEM. Mann-Whitney U test was used for testing the difference between healthy donors and sepsis patients. Bold values for p<0.05

**Supplementary Table 3. Cytokines in serum samples of survivors and non-survivors.**

| Variables |  | D1 | |  | D3 | |  | D5 | | |  | D6 | | |  | D7 | |
| --- | --- | --- | --- | --- | --- | --- | --- | --- | --- | --- | --- | --- | --- | --- | --- | --- | --- |
|  |  | cytokines | *p* value |  | cytokines | *p* value |  | cytokines | | *p* value |  | cytokines | | *p* value |  | cytokines | *p* value |
| IL-1β | S | 18.31±9.16 | 0.8371 |  | 10.44±4.912 | 0.9406 |  | 1.968±0.2244 | 0.7193 | |  | 1.663±0.2593 | 0.5119 | |  | 1.382±0.1342 | 0.2249 |
|  | NS | 15.22±5.745 |  |  | 10.99±5.25 |  |  | 1.82±0.3648 |  |  |  | 1.735±0.2513 |  |  |  | 1.667±0.1842 |  |
| IL-18 | S | 1381±664.3 | 0.9857 |  | 3709±2836 | 0.3753 |  | 3071±2036 | 0.8034 | |  | 727.9±289.7 | 0.426 | |  | 1827±1327 | 0.5904 |
|  | NS | 1402±714.8 |  |  | 1097±352.6 |  |  | 2289±1811 |  |  |  | 685.3±203.2 |  |  |  | 860.6±315.8 |  |
| TNF-a | S | 8.411±4.464 | 0.4884 |  | 12.03±10.12 | 0.5126 |  | 1.431±0.1395 | 0.0979 | |  | 1.294±0.16 | 0.3343 | |  | 1.425±0.3686 | 0.4841 |
|  | NS | 3.427±0.9242 |  |  | 4.997±2.874 |  |  | 1.082±0.06804 |  |  |  | 1.012±0.04238 |  |  |  | 1.077±0.05812 |  |
| MCP-1 | S | 3674±818.5 | 0.099 |  | 1533±311 | 0.08 |  | 753.8±152.6 | 0.1978 | |  | 1020±245.4 | 0.3254 | |  | 1117±331.8 | 0.6469 |
|  | NS | 7980±3447 |  |  | 2576±436 |  |  | 1055±107.4 |  |  |  | 1000±119.1 |  |  |  | 1328±158.1 |  |
| IFN-r | S | 50.93±34.38 | 0.121 |  | 56.57±29.33 | 0.7028 |  | 3.53±0.965 | 0.779 | |  | 5.421±2.958 | 1 | |  | 4.524±1.942 | 0.6865 |
|  | NS | 1253±1197 |  |  | 81.23±54.99 |  |  | 3.957±0.9566 |  |  |  | 4.2±2.094 |  |  |  | 5.868±2.702 |  |
| IL-6 | S | 5129±2790 | 0.0564 |  | 200.9±36.81 | 0.3602 |  | 159.3±46.11 | 0.3431 | |  | 199.5±50.49 | 0.9278 | |  | 148.1±65.37 | 0.1183 |
|  | NS | 21531±11040 |  |  | 1637±1426 |  |  | 248.7±89.87 |  |  |  | 177.9±44.26 |  |  |  | 432.7±195.9 |  |
| IL-12p70 | S | 15.08±9.783 | 0.6974 |  | 13.86±7.495 | 0.8226 |  | 1.422±0.1599 | 0.468 | |  | 1.538±0.1949 | 0.5423 | |  | 1.383±0.1537 | 0.9989 |
|  | NS | 8.903±4.439 |  |  | 11.58±6.596 |  |  | 1.252±0.08754 |  |  |  | 1.255±0.0754 |  |  |  | 1.383±0.1501 |  |
| IL-8 | S | 338.7±73.36 | 0.103 |  | 93.6±16.64 | 0.226 |  | 69.44±18.61 | 0.0552 | |  | 97.49±21.79 | 0.0678 | |  | 101.6±23.69 | 0.052 |
|  | NS | 3981±3445 |  |  | 164.1±49.51 |  |  | 146.5±37.44 |  |  |  | 121.8±6.962 |  |  |  | 183±29.73 |  |
| IL-10 | S | 58.12±13.98 | **0.0203** |  | 22.68±5.896 | 0.2943 |  | 16.19±8.269 | 0.7761 | |  | 14.99±6.425 | 0.426 | |  | 13.78±5.232 | 0.3942 |
|  | NS | 486.6±273.7 |  |  | 48.57±21.31 |  |  | 19.86±7.822 |  |  |  | 23.84±9.421 |  |  |  | 25.13±14.49 |  |

Variables are described as mean±SEM.

S, survivors; NS, non-survivors; SEM, Standard Error of Mean. D1, day 1 after the onset of sepsis; D3, day 3 after the onset of sepsis……

The n value of survivors on D1, D3, D5, D6 and D7 was 17, 16, 12, 11 and 10 respectively;

The n value of non-survivors on D1, D3, D5, D6 and D7 was 7, 6, 5, 7 and 6 respectively.

Bold values for p<0.05

**Supplementary Table 4. Demographics and clinical characteristics of the 18 patients**

| Variables | All patients n=18 | D6 ASC-Speck high n=11 | D6 ASC-Speck low n=7 | p value |
| --- | --- | --- | --- | --- |
| Demographics |  |  |  |  |
| Age(years) | 67.50(59.25-77.00) | 64.00(56.00-76.50) | 71.00(61.50-82.00) | 0.260 |
| Weight(kg) | 75.00(65.00-80.25) | 75.00(71.00-78.50) | 65.00(61.50-81.00) | 0.664 |
| Height(cm) | 165.50(162.25-179.50) | 168.00(162.50-179.00) | 165.00(162.50-175.50) | 0.892 |
| Gender(male) | 9(50.00%) | 5(45.45%) | 4(57.14%) | 1.000 |
| Site of infection |  |  |  |  |
| Lung | 3(16.67%) | 3(27.27%) | 0(0%) | 0.245 |
| Abdomen | 13(72.22%) | 8(72.73%) | 5(71.43%) | 1.000 |
| Genitourinary tract | 1(5.56%) | 0(0%) | 1(14.29%) | 0.389 |
| Catheter | 1(5.56%) | 0(0%) | 1(14.29%) | 0.389 |
| Mediastinum | 2(11.11%) | 2(18.18%) | 0(0%) | 0.497 |
| Combined | 2(11.11%) | 2(18.18%) | 0(0%) | 0.497 |
| Culture positive | 17(94.44%) | 10(90.91%) | 7(100%) | 1.000 |
| Organism |  |  |  |  |
| Bacteria | 16(88.89%) | 9(81.82%) | 7(100%) | 0.497 |
| Gram-negative | 10(55.56%) | 4(36.36%) | 6(85.71%) | 0.066 |
| Gram-positive | 11(61.11%) | 7(63.64%) | 4(57.14%) | 1.000 |
| Fungal | 5(27.78%) | 4(36.36%) | 1(14.29%) | 0.596 |
| Viral | 0(0%) | 0(0%) | 0(0%) |  |
| Mixed | 8(44.44%) | 4(36.36%) | 4(57.14%) | 0.631 |
| Septic shock | 15(83.33%) | 10(90.91%) | 5(71.43%) | 0.528 |
| 14-day mortality | 1(5.56%) | 0(0%) | 1(14.29%) | 0.389 |
| 28-day mortality | 3(16.67%) | 1(9.09%) | 2(28.57%) | 0.528 |
| 90-day mortality | 6(33.33%) | 1(9.09%) | 5(71.42%) | **0.013** |
| Clinical parameters |  |  |  |  |
| PCT(ng/ml) D1 or D2 | 7.44(2.63-39.72) | 8.65(3.36-32.55) | 3.50(1.72-55.85) | 0.786 |
| WBC(10^9^/L) D1 | 15.93(8.77-24.40) | 16.90(9.13-31.58) | 14.96(9.12-18.81) | 0.235 |
| WBC (10^9^/L) D2 | 12.40(9.42-30.10) | 14.13(10.38-32.11) | 9.75(9.07-19.08) | 0.319 |
| WBC (10^9^/L) D3 | 11.65(9.69-25.48) | 13.30(9.46-22.00) | 11.62(10.37-20.41) | 0.928 |
| WBC (10^9^/L) D4 | 11.48(9.93-18.36) | 11.15(10.36-17.52) | 11.80(9.09-17.09) | 0.651 |
| WBC (10^9^/L) D5 | 12.14(10.32-21.22) | 12.21(10.62-24.57) | 10.50(8.68-17.59) | 0.277 |
| WBC (10^9^/L) D6 | 14.58(11.21-20.88) | 16.44(13.4-28.78) | 12.59(8.29-17.35) | 0.124 |
| WBC (10^9^/L) D7 | 14.04(11.09-19.84) | 15.81(13.40-23.68) | 11.04(7.83-12.81) | 0.088 |
| CRP(mg/l)D1 | 167.55(93.50-256.48) | 179.30(147.75-287.00) | 98.90(77.30-206.60) | 0.151 |
| CRP(mg/l)D2 | 219.10(162.78-274.85) | 219.60(218.75-321.75) | 159.40(102.25-210.90) | **0.031** |
| CRP(mg/l)D3 | 190.15(143.73-259.80) | 202.10(176.15-267.25) | 153.40(137.85-174.05) | 0.174 |
| CRP(mg/l)D4 | 150.45(107.10-186.18) | 156.00(110.10-196.15) | 148.30(104.90-154.15) | 0.709 |
| CRP(mg/l)D5 | 107.10(74.25-145.38) | 109.90(79.75-142.05) | 104.30(77.30-134.85) | 0.790 |
| CRP(mg/l)D6 | 82.80(54.28-116.08) | 100.80(54.85-125.20) | 64.80(55.35-110.40) | 0.435 |
| CRP(mg/l)D7 | 99.05(53.25-126.95) | 126.20(61.65-157.60) | 74.40(55.90-103.10) | 0.120 |
| LDH(U/l)D1 | 284.00(235.25-466.00) | 460.00(260.00-670.50) | 244.00(219.00-292.00) | **0.026** |
| LDH(U/l)D2 | 291.50(216.50-453.75) | 327.00(208.00-420.00) | 256.00(235.50-472.50) | 0.651 |
| LDH(U/l)D3 | 260.50(215.25-416.25) | 277.00(221.50-384.50) | 244.00(215.00-399.50) | 1.000 |
| LDH(U/l)D4 | 253.50(214.75-355.50) | 325.00(217.50-347.00) | 253.00(211.50-315.50) | 0.786 |
| LDH(U/l)D5 | 314.50(238.50-361.75) | 337.00(256.50-380.00) | 246.00(209.50-349.50) | 0.383 |
| LDH(U/l)D6 | 331.00(249.50-388.75) | 385.00(286.50-425.50) | 263.00(210.00-354.50) | 0.116 |
| LDH(U/l)D7 | 310.00(269.75-361.00) | 324.00(303.50-392.50) | 284.00(235.00-301.50) | 0.105 |
| Lactate(mg/dl)D1 | 19.55(12.93-47.80) | 19.20(12.45-48.65) | 20.80(18.30-38.25) | 0.684 |
| Lactate(mg/dl)D2 | 16.40(10.30-33.10) | 11.45(9.60-29.98) | 19.60(15.35-27.75) | 0.270 |
| Lactate(mg/dl)D3 | 15.00(9.45-18.10) | 10.50(8.65-19.45) | 15.60(12.95-17.25) | 0.925 |
| Lactate(mg/dl)D4 | 12.00(10.40-18.28) | 11.60(10.15-15.75) | 15.10(10.85-18.05) | 0.446 |
| Lactate(mg/dl)D5 | 10.55(9.23-16.48) | 10.40(9.25-14.50) | 10.80(9.10-16.55) | 0.442 |
| Lactate(mg/dl)D6 | 10.95(9.25-14.73) | 10.70(8.75-13.40) | 12.20(9.90-15.30) | 0.663 |
| Lactate(mg/dl)D7 | 10.60(8.70-14.58) | 11.50(9.05-14.65) | 9.70(8.35-13.20) | 0.954 |
| Scoring models |  |  |  |  |
| SOFA(D1) | 13.50(10.25-14.00) | 14.00(11.50-14.00) | 11.00(8.50-14.50) | 0.517 |
| SOFA(D2) | 11.00(9.00-15.75) | 10.00(9.00-14.50) | 12.00(9.00-16.50) | 0.663 |
| SOFA(D3) | 10.50(8.00-15.50) | 10.00(8.00-14.00) | 11.00(8.50-16.50) | 0.673 |
| SOFA(D4) | 9.00(7.25-13.00) | 9.00(8.00-12.50) | 9.00(6.50-15.00) | 0.901 |
| SOFA(D5) | 8.00(6.00-11.50) | 8.00(7.00-10.00) | 7.00(5.00-14.50) | 0.899 |
| SOFA(D6) | 9.00(6.25-10.75) | 9.00(7.50-10.00) | 6.00(4.00-13.50) | 0.971 |
| SOFA(D7) | 8.00(4.25-9.50) | 8.00(6.50-8.00) | 4.00(2.50-14.50) | 0.892 |
| SAPSⅡ(D1) | 70.00(52.00-78.75) | 70.00(58.50-79.50) | 65.00(48.50-77.50) | 0.569 |
| SAPSⅡ(D2) | 69.50(48.50-76.50) | 64.00(48.00-78.50) | 75.00(60.00-76.00) | 0.483 |
| SAPSⅡ(D3) | 62.00(48.25-69.25) | 60.00(47.00-66.50) | 67.00(49.50-75.00) | 0.494 |
| SAPSⅡ(D4) | 58.00(43.25-71.75) | 56.00(50.00-71.50) | 60.00(41.00-77.50) | 0.847 |
| SAPSⅡ(D5) | 49.50(39.25-66.00) | 51.00(40.50-61.50) | 48.00(35.00-79.50) | 0.486 |
| SAPSⅡ(D6) | 48.50(35.75-64.00) | 49.00(44.50-60.50) | 38.00(32.00-78.50) | 0.797 |
| SAPSⅡ(D7) | 47.50(38.25-65.50) | 48.00(44.00-65.00) | 38.00(29.50-75.50) | 0.965 |
| APACHEⅡ(D1) | 30.50(24.75-35.25) | 31.00(25.50-32.50) | 30.00(26.00-36.00) | 0.916 |
| APACHEⅡ(D2) | 31.50(25.75-34.00) | 33.00(26.50-34.00) | 31.00(24.00-34.00) | 0.560 |
| APACHEⅡ(D3) | 30.00(20.00-35.50) | 31.00(24.50-35.00) | 24.00(19.00-32.00) | 0.452 |
| APACHEⅡ(D4) | 26.00(21.25-30.75) | 26.00(22.50-31.00) | 26.00(17.50-29.50) | 0.340 |
| APACHEⅡ(D5) | 25.50(19.25-28.75) | 25.00(19.50-28.00) | 26.00(18.50-30.00) | 0.845 |
| APACHEⅡ(D6) | 22.00(21.00-29.00) | 22.00(21.00-29.00) | 22.00(14.00-29.00) | 0.525 |
| APACHEⅡ(D7) | 23.50(18.25-30.75) | 24.00(19.50-29.50) | 23.00(14.50-30.50) | 0.581 |

Variables were shown as frequencies (percentages) for categorical data and medians (interquartile ranges) for continuous data.

D1 mean the first day after onset of spesis, D2 means the second day after onset of sepsis……

Bold values for p<0.05

**Supplementary Table 5. ROC curve analysis established the best cutoff values for different parameters to predict 90-day mortality.**

| Variables | Cutoff | Sensitivity% | Specificity% | AUROC | 95% CI | p value |
| --- | --- | --- | --- | --- | --- | --- |
| mASC(absolute number) | 1650（/ml） | 83.33 | 83.33 | 0.875 | 0.699-1.05 | **0.011** |
| mASC(%) | 0.1994（%） | 66.67 | 83.33 | 0.778 | 0.552-1.003 | 0.061 |
| [SOFA](file:///B:\writing\6%20complete\Supplementary%20table%202.3.4\table%202.6（final%20version）.xlsx#RANGE!A1) | 6.500 | 16.67 | 100.00 | 0.507 | 0.185-0.829 | 0.963 |
| [SAPSⅡ](file:///B:\writing\6%20complete\Supplementary%20table%202.3.4\table%202.6（final%20version）.xlsx#RANGE!A1) | 70.50 | 66.67 | 66.67 | 0.556 | 0.256-0.855 | 0.708 |
| [APACHEⅡ](file:///B:\writing\6%20complete\Supplementary%20table%202.3.4\table%202.6（final%20version）.xlsx#RANGE!A1) | 34.50 | 50.00 | 83.33 | 0.569 | 0.263-0.876 | 0.640 |
| [Weight](file:///B:\writing\6%20complete\Supplementary%20table%202.3.4\table%202.6（final%20version）.xlsx#RANGE!A1) | 66.00（kg） | 66.67 | 83.33 | 0.674 | 0.355-0.993 | 0.242 |
| [Age](file:///B:\writing\6%20complete\Supplementary%20table%202.3.4\table%202.6（final%20version）.xlsx#RANGE!A1) | 78.00（years） | 50.00 | 91.67 | 0.632 | 0.344-0.92 | 0.374 |
| [IFN-γ](file:///B:\writing\6%20complete\Supplementary%20table%202.3.4\table%202.6（final%20version）.xlsx#RANGE!A1) | 3.180(pg/ml) | 100.00 | 41.67 | 0.681 | 0.408-0.954 | 0.223 |
| [IL-10](file:///B:\writing\6%20complete\Supplementary%20table%202.3.4\table%202.6（final%20version）.xlsx#RANGE!A1) | 222.7(pg/ml) | 33.33 | 100.00 | 0.556 | 0.242-0.869 | 0.708 |
| [IL-12p70](file:///B:\writing\6%20complete\Supplementary%20table%202.3.4\table%202.6（final%20version）.xlsx#RANGE!A1) | 1.060(pg/ml) | 100.00 | 25.00 | 0.556 | 0.281-0.830 | 0.708 |
| IL-18 | 331.2(pg/ml) | 83.33 | 66.67 | 0.611 | 0.309-0.914 | 0.454 |
| IL-1β | 6.840(pg/ml) | 66.67 | 66.67 | 0.583 | 0.303-0.864 | 0.574 |
| IL-6 | 53952(pg/ml) | 33.33 | 100.00 | 0.583 | 0.283-0.884 | 0.574 |
| IL-8 | 99.80(pg/ml) | 100.00 | 25.00 | 0.583 | 0.297-0.870 | 0.574 |
| MCP-1 | 3179(pg/ml) | 50.00 | 66.67 | 0.542 | 0.249-0.834 | 0.779 |
| TNFα | 1.085(pg/ml) | 100.00 | 25.00 | 0.514 | 0.243-0.784 | 0.925 |

AUC, area under the ROC curve; CI, confidence interval; mASC(absolute number), absolute number of ASC-speck+ monocytes; mASC(%), the percentage of ASC-specks in monocytes. Bold values for *p*<0.05.

*mASC(absolute number) and mASC(%) values are from day 6 after onset of sepsis, values of all the other variables are from day 1 after onset of sepsis.
